# Obliteration of the Inferior Vena Cava

**Published:** 1841-08

**Authors:** 


*Obliteration of the Inferior Vena Cava.*—Dr. Gely has recorded, in a recent number of the *Journal de la Section de Medicine de la Societe Academique du Departement de la Loire-Inferieure,* the following interesting case of obliteration of the vena cava.

Gerard, a seaman, 48 years of age, strong constitution, sanguineous temperament, was admitted towards the close of the winter of 1838, into the hospital of Nantes, with symptoms of anasarca, ascites, febrile oppression, extreme varicose dilatation of the veins of the lower extremities, and of the abdomen, which were also excoriated over several spots. He stated that the varicose state of the veins came on during the winter of 1823, when he had the misfortune to have his legs frozen. He however recovered so far as to be able again to go to sea, where he contracted one of those cutaneous affections so common amongst the negroes. Ulcers and abscesses formed on various parts of his legs, and he was long confined to bed; the varicose state of the vessels increased; and, for the last five or six years, he had been unfit for work. After a residence of a few weeks in the hospital, he died, with all the symptoms of some serious affection of the circulatory organs.

On dissection the superficial veins of the lower extremities presented the appearance of thick cords, doubled on themselves a great number of times, in the same way, indeed, as the *vas deferens* is at its origin. This mass, as it approached the crural arch, increased in volume; but after passing this, in its progress over the abdomen, it diminished in bulk till it reached the false ribs. The varicose veins were perceptibly larger and more flexuous on the right than on the left side. The varicose vessels over the trunk of the body were the abdominal tegumentary vessels, which anastomosed on each side with a large external mammary branch, which terminated in the axilla. The femoral and iliac veins of the right side were filled with false membranes to such an extent as to diminish the calibre of the venous canal to a tenth of its natural diameter. The false membranes became more numerous as they approached the *vena cava*, which was reduced to the state of a cartilaginous cord, as far as the point where the emulgient veins unite with it, above which portion it was pervious, but reduced in diameter.

The right epigastric vein was somewhat narrowed in diameter, but that of the left side was enormously dilated, ascended towards the umbilicus, coursed along the suspensory ligament of the liver, and followed exactly the course of the umbilical vein. The two renal veins were very much dilated, and on the right side an abnormal branch opened into the *vena cava*, and was continuous with the *vena comes* of the crural nerve, which was much dilated, and seemed to receive the blood from the deep vessels of the thigh.

The heart was hypertrophied, with dilation of all its cavities; and osseous concretions were met with around the aortic and auriculo-ventricular orifices, as well as in the coats of the aorta.

This case is extremely curious, as showing the mode in which the venous circulation had been kept up in spite of the destruction of the canal of the *vena cava*. Thus, on the right side, the blood from the lower extremity, collected by the superficial veins, passed along the tegumentary veins and external mammary into the axillary vein, and from that into the superior cava; whilst the blood of the deep portions of the same limb was emptied into the *vena comes* of the saphena nerve; and from it into the *vena cava* inferior, above the contracted portion, and lumbo-vertebral venous plexus. On the left side, again, the superficial blood returned to the centre of circulation in the same manner as that on the right side; whilst the venous blood from the deep portions of the limb was poured into the epigastric vein, and from it into the sinus of the *vena portæ* by the umbilical vein.

The opening of one of the epigastric veins into the umbilical vein, and the enormous dilation of the vein which accompanies the crural nerve, and communicated with the *vena cava* inferior, were the two most striking anomalies in this case.—*Gazette Med. de Paris, Nov.* 7, 1841.

